# Epithelial Cell Culture from Human Adenoids: A Functional Study Model for Ciliated and Secretory Cells

**DOI:** 10.1155/2013/478713

**Published:** 2013-02-05

**Authors:** Claudia González, Marisol Espinosa, María Trinidad Sánchez, Karla Droguett, Mariana Ríos, Ximena Fonseca, Manuel Villalón

**Affiliations:** ^1^Department of Otorhinolaryngology, Faculty of Medicine, Pontificia Universidad Católica de Chile, Marcoleta 350, 2°Piso, Santiago 8330033, Chile; ^2^Department of Physiology, Faculty of Biological Sciences, Pontificia Universidad Católica de Chile, Alameda 340, Santiago 8331150, Chile

## Abstract

*Background*. Mucociliary transport (MCT) is a defense mechanism of the airway. To study the underlying mechanisms of MCT, we have both developed an experimental model of cultures, from human adenoid tissue of ciliated and secretory cells, and characterized the response to local chemical signals that control ciliary activity and the secretion of respiratory mucins *in vitro*. *Materials and Methods*. In ciliated cell cultures, ciliary beat frequency (CBF) and intracellular Ca^2+^ levels were measured in response to ATP, UTP, and adenosine. In secretory cultures, mucin synthesis and secretion were identified by using immunodetection. Mucin content was taken from conditioned medium and analyzed in the presence or absence of UTP. *Results*. Enriched ciliated cell monolayers and secretory cells were obtained. Ciliated cells showed a basal CBF of 10.7 Hz that increased significantly after exposure to ATP, UTP, or adenosine. Mature secretory cells showed active secretion of granules containing different glycoproteins, including MUC5AC. *Conclusion*. Culture of ciliated and secretory cells grown from adenoid epithelium is a reproducible and feasible experimental model, in which it is possible to observe ciliary and secretory activities, with a potential use as a model to understand mucociliary transport control mechanisms.

## 1. Introduction

Mucosa of the nasal cavity, like the rest of the airway, is covered by a ciliated pseudostratified epithelium, which is composed of ciliated and nonciliated columnar cells covered with microvilli, basal cells, and secretory cells. Ciliated and secretory cells compose the functional unit responsible for mucociliary clearance. The synchronous movement of cilia with the subsequent displacement of the mucus layer containing dust, bacteria, and other elements into the pharynx is the basic defense mechanism of the airway [1]. Mucociliary transport (MCT) velocity is determined by several factors, such as ciliary beat frequency (CBF), rate of mucus secretion and rheological properties of mucus.

 Normal mucociliary clearance requires three essential elements: active cilia, mucus, and periciliar fluid [1]. Respiratory cilia beat in a synchronous pattern. The energy required for ciliary movement derives from adenosine 5′-triphosphate (ATP) hydrolyzed by the ATPase activity of the dynein arms within cilia. Basal CBF in humans varies between 9 and 15 Hz [2]. The activation of purinergic receptors by ATP or UTP induces a significant increase in CBF in ciliated cells and mucus secretion in secretory cells from different species [3–7]. The response to ATP and its metabolites appears to be important in the airway [8–10]. Also the ATP increases CBF, coupled to an increase in the intracellular calcium concentration ([Ca^2+^]_*i*_) in tracheal and oviductal ciliary cells [11, 12]. Therefore, systematic analysis of the response of ciliary activity and mucus secretion to ATP, and other purinergic agonists, like UTP or adenosine, is a suitable model to characterize and evaluate these cells' function. 

Mucus is made of material produced by secretory cells and submucosal glands and is composed, primarily, of water (95%), inorganic salts, and macromolecules such as glycoproteins (mucins), lipids, and others (5%) [13]. Mucins provide the viscoelastic characteristics to the mucus. The most abundant mucins that form the gel in airway are MUC5AC and MUC5B. Also, there are membrane-bound mucins such as MUC1, MUC4, and MUC16 [14–16]. Immunostaining studies in normal airways have shown that secretory cells predominantly produce MUC5AC. In addition, MUC5B is synthesized mainly, but not exclusively, in goblet cells of submucosal glands [16].

To date, it has not been possible to obtain immortalized ciliated respiratory cell lines, so most ciliary functional studies have been developed in ciliated cell cultures or explants of ciliated epithelium *in vitro*. Airway-liquid interface (ALI) ciliated cell cultures have been developed out of epithelial samples from nasal mucosa (nasal septum, turbinates, nasal polyps) and trachea [17–19]. Explants of epithelium preserved in saline solution have been used for CBF studies in short periods of time [20–22]. In addition, adenoid tissue has also been used to understand the conditions that are required in order to foster differentiation into ciliated and mucus producing cells [23].

Adenoidectomy with or without tonsillectomy is one of the most common surgeries in otolaryngology, where the adenoids are usually discarded. Moreover, a large percentage of pediatric patients who undergo these interventions are generally healthy children. Consequently, given both the easy access to sample procurement, the large sample size-compared with tissue obtained from turbinates and nasal polyps and the prevalence of healthy patients, the adenoid tissue possess numerous advantages as a source of further development of an experimental model for ciliary function studies. Furthermore, the presence of secretory cells in adenoids, with the most abundant mucins in the airway MUC5AC and MUC5B, makes this an attractive model for *in vitro* studies of the airways.

Our objective was to develop and evaluate the functionality of a reproducible experimental model that allows for studies of both ciliated and secretory cells, from human adenoid epithelium explants, in order to better understand the mechanisms underlying mucociliary transport in the human airway.

## 2. Material and Methods

### 2.1. Tissue Samples

Adenoid tissue was obtained from pediatric patients, aged between 3 and 12 years, undergoing adenoidectomy for obstructive pathology (adenoid or adenotonsillar hypertrophy) with parental informed consent. The study design and informed consent were reviewed and approved by the Ethics Committee of Pontificia Universidad Católica de Chile.

Immediately after procurement, adenoid tissue was placed in a physiological saline solution, so as to remove blood clots, and later in a Hank's balanced salt solution (HBSS; Sigma-Aldrich H1387) pH 7.4, supplemented with antibiotics (10 **μ**g/mL streptomycin, 100 U/mL penicillin G, 0.125 **μ**g/mL amphotericin B; Gibco BRL U.S.A).

Adenoid tissue samples were washed with DMEM/F12 (Invitrogen Corp. NY; USA) supplemented with antibiotics to remove traces of blood. The tissue was then placed in a DMEM/F12 medium with antibiotics and pronase (P5147; Sigma Chemical Co., St. Luis, MO, USA) 0.05% w/v and left overnight at 4°C. The next day, the tissue was transferred into a container with DMEM-F12 and 5% bovine fetal serum (FBS; Biological Industries, Israel). The epithelium was mechanically removed by using fine point Dumont forceps and student vannas scissors; at this point, samples were separated for two protocols: ciliated cell culture and secretory cell culture.

#### 2.1.1. Ciliated Cell Culture

Cultures were prepared following the method introduced by Verdugo et al. [24], to yield a confluent monolayer of epithelial cells [25]: epithelium is cut into 2–4 mm pieces, which are washed with sterile HBSS and soaked in NHS (137 mM NaCl; 5.09 mM KCl; 1.14 mM Na_2_HPO_4_  ×  2H_2_O; 0.18 mM KH_2_PO_4_; 0.923 mM MgCl_2_  ×  6H_2_O; 0.91 mM CaCl_2_  ×  2H_2_O; 4.07 mM NaHCO_3_; 21.5 mM glucose, pH 7.4), supplemented with 1% of vitamins, 1% essential amino acids, 1% non-essential amino acid, 1% pyruvate and antibiotics: 0.2 mg/mL neomycin and 0.12 mg/mL penicillin (all these reagents: Invitrogen Corp. NY; USA). The pieces of epithelium were placed onto a coverslip pre-treated with 0.1% gelatin (G9391, Sigma Chemical Co, St Louis, MO, USA) in Rose chambers. Explants were covered with a sterile dialysis membrane,—(Spectra/Por∗2, MWCO 12–14,000; number 25218-468, VWR Scientific) pre-hydrated with distilled water. Rose chambers were filled with 2 mL of NHS medium, containing 10% heat inactivated horse serum (Biological Industries, Israel) (pH 7.2–7.4) and kept in an incubator at 37°C. The culture medium was renewed every 48 h. A culture was ready to be used when a monolayer of ciliated cells showed spontaneous ciliary activity.

From each adenoid sample, we obtained around 6 cultures, each one with 4 or 5 explants surrounded by a monolayer of ciliated cells. For the purpose of this study, we used 48 cultures of ciliated cells, obtained from 22 patients. An estimate success rate fluctuates between 80 and 100%.

*CBF Measurements.* CBF was recorded using a microphotodensitometric technique [26] Nikon-Diaphot-inverted-microscope-mounted, according to the procedure previously described [25]. Basal CBF was measured every minute over five minutes. If the frequency remained constant, additional experiments were conducted.

Cultures were washed three times with HBSS, and then equilibrated at 35°C for a period of 15 min. Spontaneous CBF was measured for 5 min before being treated with ATP, UTP and Adenosine 1 **μ**M, 10 **μ**M, 50 **μ**M, or 100 **μ**M. After 25 min of CBF measurements, cultures were washed three times with HBSS to completely remove the treatment and observe recovery of the spontaneous activity. 

*Intracellular Calcium ( *[*Ca* ^2+^]_*i*_ *) Levels Measurements. *[Ca^2+^]_*i*_ was determined using a spectrofluorometric technique described previously [27]. Cultures of ciliated cells were loaded with 1.5 **μ**M Fura-2AM (Invitrogen) for 1 h at 37°C. The fluorescence was observed at room temperature with an Olympus fluorescence microscope coupled to an image acquisition system (Metafluor, Universal Imaging Corporation, v6.1). Images were acquired at excitation wavelength of 340 and 380 nm and detected at 510 nm, which were analyzed with the Metamorph program (Universal Imaging Corporation, v6.1).

*Scanning Electron Microscopy (SEM).* SEM was done on ciliated cell culture at 9 days. The cells were fixed with 2% PFA in PBS, pH 7.2, for 45 min at 4°C. Then, tissue was washed, dehydrated in a progression of increasing ethanol concentrations, up to 100% ethanol, and critical point dried (CPDS Model-2002). Samples were sputter-coated with gold palladium using the Varian/Vacuum equipment Evaporator division PS 10E and observed with a JEOL JSM-25 S II Scanning Microscope.

#### 2.1.2. Secretory Cell Culture

A total of 98 adenoids were used as samples. The average age of the donor patients was 5.16 ± 2.7 years old, ranging from 2 to 14 years.

The layers of the epithelium, obtained as previously described, were transferred into a tube where they were dispersed mechanically by forcing them through a pipette. Finally, the cellular suspension was centrifuged at 300 ×g for 5 min. The supernatant was discarded and the pellet was resuspended in a DMEM-F12 medium supplemented with antibiotics and 5% FBS.

The cells were seeded in two different culture systems: (1) in 50 mL flasks and (2) in wells of a 4-well plate (Nunc, NY, USA) with inserted coverslips (12 mm diameter), previously bathed with collagen 10 *μ*g/cm^2^ type I (in order to favor cellular adhesion). From one adenoid sample, we obtained 4 cultures in flasks and one 4-well plate, with a success rate of 100%.

 Approximately, 1 × 10^6^ cells/mL were seeded in 50 mL flasks (1.5 mL of cellular suspension) and 10 times less in the wells (300 *μ*L of the cellular suspension, per well). On the third day of culture, the culture medium was changed for the first time for fresh medium, which contained a DMEM/F12 (1 : 1) culture medium with antibiotics and later supplemented with the following components: 5 *μ*g/mL insulin (Serological Corporation), 5 *μ*g/mL transferrin (Invitrogen), 10 *μ*g/mL EGF (Upstate), 0.1 *μ*M dexametasone (Sigma-Aldrich), 10 *μ*g/mL cholera toxin (Sigma-Aldrich), 15 *μ*g/mL bovine hypophysis extract (Invitrogen) and 1 *μ*M retinol (Sigma-Aldrich) at a pH of 7.4 [28]. Because of the chemical properties of the retinol, it was added, at the same time that the supplement, procedure that was in darkness. The culture medium was changed every other day, at which point each flask was washed with 2 mL of base culture medium and each well was washed with 1 mL of the same, for approximately two to three minutes. Later, the cells were left in a supplemented culture medium for 48 more h. Finally, the cultures were incubated in a controlled atmosphere of 5% CO_2_ and 95% O_2_ at 37°C. 

*Immunofluorescence*. Identification of mucins in cell culture monolayer: cells grown on coverslips were fixed with paraformaldehyde 2% (PFA) in 10 mM phosphate buffered saline (PBS), pH 7.4 for 10 min at room temperature. The PFA was prepared fresh as follows: PFA was dissolved in water between 55–60°C. When it was cold, a stock of modified Ca^2+^-free Hank's solution (“Small buffer” 1X: 137 mM NaCl, 5 mM KCl, 1.1 mM Na_2_HPO_4_, 0.4 mM KH_2_PO_4_, 4 mM NaHCO_3_, 5.5 mM glucose, 2 mM MgCl_2_, 2 mM EGTA, 5 mM PIPES, pH 7.2) with 0.1% Triton X-100 was added [29]. 

 After fixation, cells were nonpermeabilized or permeabilized with 0.5% Triton X-100 in “Small Buffer” mode for 10 min at room temperature and then, non-specific binding was blocked with bovine serum albumin (BSA, Sigma-Aldrich) free of immunoglobulins in “Small buffer”, for 45 min at 4°C. Slides of non-permeabilized or permeabilized cells were incubated with polyclonal antibodies against MUC5AC: MAN5AC 1 : 2000 [14] and MUC5AC 1 : 600 (gift from Dr. Ho) [30]; and monoclonal antibody against MUC5B: EU5B 1 : 2000 [31], during overnight at 4°C. After washing with “Small's Buffer” with 0.1% Tween 20-Winkler Ltda, Chile-slides were incubated with a FITC-conjugated secondary antibody or Cy2-conjugated secondary antibody for 1 h at room temperature. Slides were mounted using a drop of Fluoromount G (Electron Microscopy Sciences) and cells were visualized in a FLUOVIEW FV1000 confocal Olympus microscope. Negative controls were done without primary antibody and human cervix epithelium was used as positive control (data not shown).

 Parameters like gain, background, laser intensity, and photomultiplier were calibrated with the values of fluorescence intensity from negative controls and then the microphotographs of the positives samples were obtained.

Identificaton of mucins in tissue samples: pieces of adenoid tissue were fixed with 4% PFA in PBS pH 7.4 during 18 h at 4°C. Then, the tissue was dehydrated in alcohol, included in Paraplast (Tyco/Health Care, USA), cut in sections of 5 *μ*m each and mounted on Xylane-(3-aminopropyl) triethoxysilane, Sigma-Aldrich-treated slides to foster adherence of tissue sections. These sections were deparaffinized and rehydrated with a gradient of ethanol and rinsed in 10 mM PBS, pH 7.4. The tissue sections were blocked by means of 0.5% BSA for 45 min at room temperature. All slides were then incubated at 4°C overnight with MAN5AC antibody dissolved in PBS with 0.1% BSA. Following washing, a FITC-conjugated secondary antibody was applied to the sections for 1 h at 4°C. Cell nucleus was counterstained with propidium iodide. 

*Histochemistry.* Detection of glycosylated material in tissue samples: so as to evaluate the presence of carbohydrates associated to secretory cells of the airway epithelium, the Periodic acid Schiff (PAS) method was used. For this purpose, adenoid tissue slides were processed in the same way described previously. The tissue sections were treated with 0.5% periodic acid (Merck, Germany) for 10 min and rinsed twice with distilled water. In a dark chamber, these sections were incubated with Schiff reagent (Merck, Germany) for 30 min at room temperature. After distilled water rinsing, sections were counterstained with hematoxylin. PAS-stained sections were observed in a microscope Nikon (Optiphot-2) and captured with a digital camera (QImaging Micro Publisher 3.3 RH). 

*Collection and Concentration of Conditioned Medium Using Filtration*. Controls: conditioned media and the washing media from 18 and 21 day cultured-flasks, were collected and stored in a solution of guanidinium chloride (GdmCl) (Sigma-Aldrich). The buffer contained 20 mM n-ethyl-maleimide (NEM) (Sigma-Aldrich), protease inhibitors (Roche Diagnostics) and a final concentration of 4 M GdmCl [32]. Subsequently, conditioned medias were concentrated by filtration, centrifuged at 2700 ×g at 5°C in a Centriplus cut-off 100 KDa (Amicon, MILLIPORE, USA). All samples were filtered on the same day, for periods of 30 min each. Repeated centrifugations with new volume were done in the same tube, until a reduction of 6-fold was obtained. Two fractions were obtained: hold (molecules over 100 KDa) and filtered (molecules less than 100 KDa). The final volume of concentrated mucins was 9 mL. Using dot blot, we verified the presence of mucins in each fraction. 

Treatment with UTP 0.1 mM: UTP, a purinergic agonist, is used as an inductive agent of the secretion of mucins. Cultures of 20-day growth were treated with the nucleotide UTP 0.1 mM for 22 h [33], dissolved in culture media. Culture media was conditioned for 24 h in the presence and absence of UTP (Sigma-Aldrich) and collected in GdmCl in the way described above. Conditioned media from control group correspond to a pool of *n* = 5 and from the group supplemented with UTP *n* = 4, where each n corresponds to adenoid tissue from 4 or 5 distinct patients.

*Ultracentrifugation in CsCl/GdmCl Isopycnic Gradient*. Controls: in order to analyze the mucins present in the control-conditioned media, glycoproteins were purified. The mucins were isolated from non-mucin proteins by means of a isopycnic density centrifugation. A gradient of 4 M CsCl/GdmCl density was prepared with an initial density of 1.4 g/mL CsCl as follows: 6 mL of concentrated culture media were placed in a 10 mL tube, to which 4.116 g of CsCl was added up to 10 mL, with 4 M GdmCl in 100 mM Tris-HCl, 5 mM EDTA pH 8.0. From this tube, 9 mL were transferred to a new 10 mL tube and completed up to 10 mL with GdmCl 4 M on a balance (APx-1502) up to 14 g. One mL of this solution was weighted and density was determined as 1.4 mg/mL. The remaining 9 mL were placed in a centrifuge tube of 9 mL. In the control tube the sample volume was replaced by GdmCl 4 M. Finally, tubes were centrifugated in a Beckman Rotor Type 70.1 Ti at RCF_average_ = 171.500, during 70 h at 10°C [32]. 

After centrifugation, each tube of 9 mL was emptied from above by 0.5 mL repeatedly to obtain 18 fractions. From each fraction, 10 **μ**L were analyzed with slot blot to test the presence of proteins using amido black and 100 **μ**L were tested for the presence of carbohydrates using the PAS reaction. Also, we demonstrated the presence of mucins forming the gel phase (MUC5AC and MUC5B) and membrane bound mucins (MUC4 and MUC16) using slot blot. We used a Metamorph 6.0 software to quantify the intensity of each band. 

Treatment with UTP 0.1 mM: conditioned media was placed in a 12 mL tube (Beckman), containing 2 mL of 1.5 g/mL CsCl, at the bottom of the tube, in buffer 0.2 M NaCl, 0.01 M EDTA, 0.01% Sodium azide, pH 7.0, and centrifuged in a Beckman SW 40 Ti rotor at RCF_average_ = 202.048 ×g during 16 : 30 h. After centrifugation, tubes were unloaded from the top by collecting fractions of 500 *μ*L. Mucins distribution were visualized by slot blot, using the antibodies MAN5AC and glycosylated material distribution was visualized by colorimetric reaction of the PAS staining and using an acid sialic lectin (Vector Laboratories, Inc. U.S.A.).

Slot Blot: A sandwich was formed using the plates from each camera (Gibco BRL U.S.A.), and placing a paper filter and a nitrocellulose membrane, previously dampened with distilled water, between the two. After adding the samples, vacuum suction was applied for 3 min. Finally, the cameras were dismounted and the nitrocellulose membranes were removed and washed in distilled water.

Detection of apomucins: the nitrocellulose membrane was washed in TBST for 5 min and blocked for 1 h with 1% milk (p/v) in a TBST buffer (10 mM Tris-HCl, 150 mM NaCl, and 0.1% (v/v) Tween 20, pH 8.0). The primary antibodies were diluted 1 : 2000 in TBST 1X and nitrocellulose membrane was exposed for 2 h. The selected polyclonal antibodies were MUC4 (MUCH4) [34] and MUC5AC (MAN5AC), and monoclonal antibodies MUC5B (EU5B) and MUC16 (CA125) [35]. Membranes were washed three times for 10 min each in TBST 1X and were exposed to the secondary antibody conjugated to peroxidase (Chemicon, Santa Cruz and Jackson) for 1 h. A chemioluminescent kit (Amersham Biosciences) was used to detect immunoreactive signals. Membranes were exposed to photographic plates (Fuji Medical X-Ray Film) and were developed automatically (AGFA, Curix60). The photographic plates were scanned and the transferred images were analyzed with densitometry, using the ImageJ program (http://rsb.info.nih.gov/ij/docs/intro.html). Using the “Analyze-Gels,” Image tool the number of pixels underneath each peak of intensity for each immunoreactive slot was calculated. This data was transferred onto a spreadsheet, where it was normalized with respect to the average value of intensity for each condition. 

Detection of glycosylated material: the nitrocellulose membrane was placed in a solution of periodic acid (1.0% (p/v), 3% acetic acid). The membrane was heated in a microwave at 50% power for 1 min. After two washes of 5 min each in distilled water, the membrane was incubated in a sodium metabisulphite solution (0.1% p/v and 1% HCl 1 N in water). Membrane was again heated in a microwave at 50% power for 30 s, and again washed twice for 5 min each with the same solution. Then, the membrane was incubated in a Schiff reactive for 10 to 30 min in darkness. The membrane was washed twice with a sodium metabisulphite solution, for 5 min each time. Finally, this membrane was washed with distilled water and left to dry, for later analysis. 

Detection of sialic acid: after the membrane was blocked, it was incubated with a biotinylated lectin (Vector Laboratories), at a concentration of 0.25 *μ*g/mL in TBST buffer. After their washing with TBST 1X, membranes were incubated for 1 h with a streptavidin-horseradish peroxidase conjugate (Amersham Laboratories, UK) diluted in blocking buffer. Labeled proteins were visualized as apomucins.

Detection of protein: Naphthol Blue Black (Sigma-Aldrich) was used for protein staining on nitrocellulose membranes. Blots were stained for 1 min and then distained for 30 minutes in 25% (v/v) isopropanol, 10% (v/v) acetic acid. Finally, the membrane was left to dry for later analysis. 

### 2.2. Data Analysis 

Data are expressed as the average ± standard error (SE). *t* test or One-way ANOVA with the Tukey's post test and graphics were performed using GraphPad Prism version 4.0 (GraphPad Software, San Diego California USA). The CBF data were analyzed after arcsin transformation [36]. The criterion for a significant difference was a final value of *P* < 0.05.

## 3. Results

### 3.1. Histology of Adenoid Epithelium Biopsy

**Figure 1:**
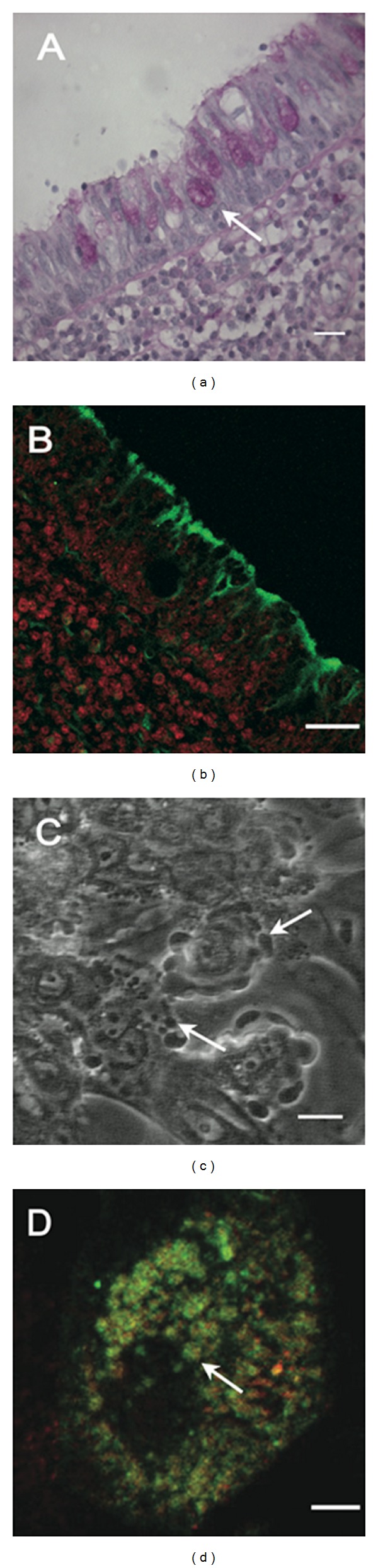
Histological sections and cultures of adenoid epithelium. Histological sections of adenoid epithelium and cultures of secretory cells. (a) Histological section of adenoid epithelium (0.5 **μ**m thick) processed to visualize intracellular sugars using a PAS method. The arrow shows the presence of PAS positive secretory material inside the cytoplasm. (b) Confocal microscopy of adenoid epithelium slides showing positive immunoreactivity for the antibody MAN5AC (green). Cell nucleus was counterstained with propidium iodide. (c) A phase contrast microscopy showing secretory cells culture after 21 days of cell growth, with plenty of secreted material attached to the cell surface over time (arrows). (d) A confocal microscopy of secretory cells showing positive immunoreactivity for MUC5AC (green) and MUC5B (red). The image corresponds to the overlapping of the two fluorescent signals. Abundant immunoreactive granular material is observed inside the cell. These observations indicate that cell culture conditions allowed for the development of cultures enriched in secretory cells from the respiratory epithelium. Bar in (a)–(c) = 20 **μ**m and (d) = 5 **μ**m.

Characterization of adenoid tissue samples using PAS/hematoxylin staining showed a columnar epithelium with secretory cells, containing PAS positive granules (Figure 1(a)). The use of the confocal microscopy demonstrated positive immunoreactivity to MUC5AC, the most common mucin in the respiratory epithelium, located in the apical end of secretory cells (Figure 1(b)). 

### 3.2. Ciliated Cell Cultures

Using a modified culture technique from Verdugo et al. [24] we were able to obtain cultures showing spontaneous ciliary beat with similar characteristics to those of the respiratory epithelium *in vivo*. 

As a result of this, a confluent monolayer of ciliated cells grew surrounding the explant. This monolayer usually stayed in place even if the explant was carefully removed from the coverslip. However, sometimes, this procedure disrupted the monolayer, so in general the explant was left in place and the measurements were performed in the monolayer away from it. Either the monolayer was formed by cell migration from the explant or by proliferating cells followed by differentiation; nevertheless, this process was not determined in this study. 

The average days needed to observe ciliary activity were 9.5 ± 2.7, ranging from 6 to 14 days. 

**Figure 2:**
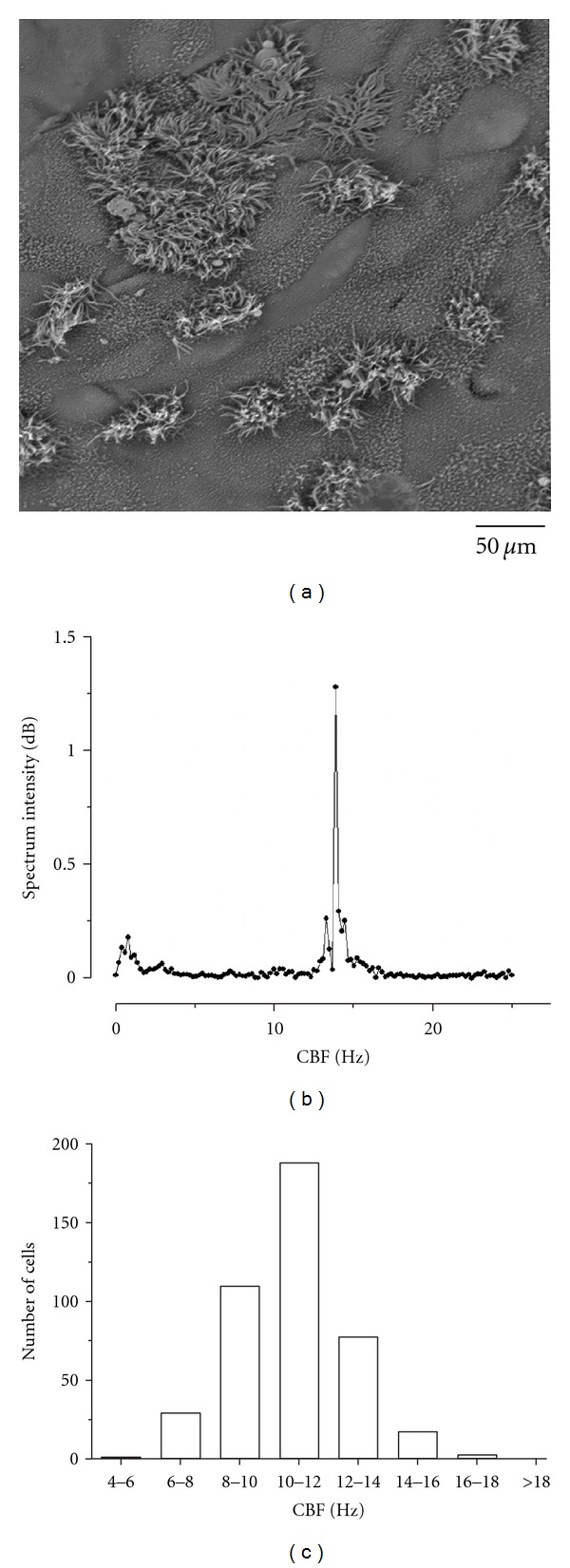
Determination of basal ciliary beat frequency (CBF). (a) Scanning electron microphotography of ciliated epithelial cells in culture. (b) Power spectrum of ciliary activity obtained from a ciliated cell in culture with a CBF peak of 13.5 Hz. (c) Distribution of basal CBF in cultures of human adenoid epithelium. Basal CBF follows a Gaussian distribution. 44.4% of the cells have a basal CBF between 10 and 12 Hz.

The results obtained by means of the scanning electron microscopy of the cultures showed monolayers of ciliated cells with cilia grouped at the center of the cell on the apical surface (Figure 2(a)).

*Detection of Ciliary Activity.* The basal CBF was measured in a total of 423 cells. Cultures showed an average CBF expressed in Hz 10.79 ± 0.09 (mean ± SEM), ranging from 5.9 to 17.5 Hz. The instant average spectrum obtained of a ciliated cell with a CBF of 13.5 Hz is shown below (Figure 2(b)).

In each culture, it was possible to observe ciliated cells with different basal CBF, showing a Gaussian distribution, according to Shapiro-Wilk test (Figure 2(c)). 44.4% of the cells had a CBF between 10 and 12 Hz. 

**Figure 3:**
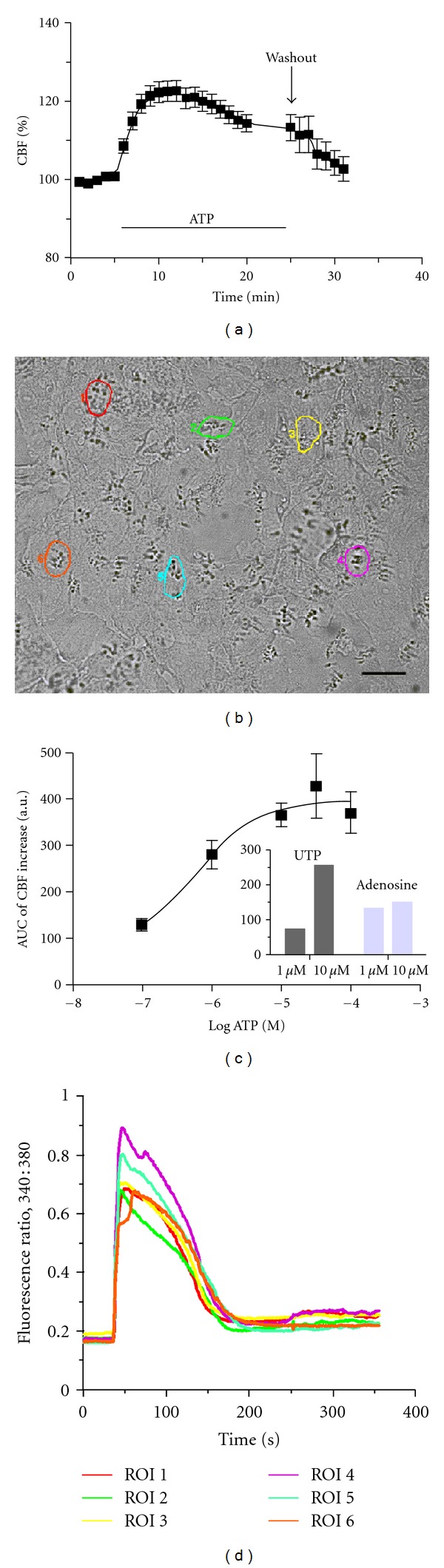
Effect of ATP, UTP, and adenosine on CBF and intracellular Ca^2+^ levels in cultured ciliated cells. (a) time course response of CBF, expressed as a percentage (%) of baseline CBF, in response to stimulation with ATP 10 *μ*M. After addition of ATP, CBF showed a rapid increase, followed by a plateau over the baseline CBF. After washing, the culture CBF returned to baseline. (b) Phase microscope image of ciliated cells in culture loaded with FURA-2AM. Showing 6 regions of interest (ROI) selected for calcium measurements with ATP stimulation. (c) ATP dose-response curve. Curve shows the logarithmic molar concentration of ATP and the area under the curve (AUC) for the CBF response observed for each concentration of ATP. Insert graph: average increase in CBF after exposure to UTP or adenosine (1 *μ*M and 10 *μ*M). (d) Time course response of intracellular calcium levels expressed as 340/380 fluorescence ratio after stimulation with ATP (10 *μ*M). After adding ATP, a rapid increase in intracellular calcium is observed in all studied ciliated cells selected previously in ROI (c).

*CBF in Response to ATP, UTP and Adenosine.* After the addition of ATP, the CBF showed a rapid increase followed by a plateau over the basal rate. When cultures were washed, CBF returned to baseline levels (Figure 3(a)). The different concentrations of ATP tested showed similar patterns in the CBF increase. The ATP response was concentration dependent, showing a maximum response to ATP of 28.7% (ATP 10 **μ**M; *n* = 9 patients), 33% (ATP 50 **μ**M; *n* = 3 patients) and 29.4% (ATP 100 **μ**M; *n* = 6 patients) (Figure 3(b)). UTP and adenosine in all concentrations induced a sustained increased in CBF (Figure 3(b)). 

*Effect of ATP on Intracellular Ca* ^2+^. Chemical signals, including purines like ATP, modify the CBF affecting intracellular calcium levels. The addition of ATP (10 **μ**M) to the cultures was observed to produce a rapid increase in the intracellular [Ca^2+^]_*i*_ concentration, expressed as the fluorescence ratio (340/380). The increase of [Ca^2+^]_*i*_ reached a peak of 4-fold higher than the baseline within 10 s. This increase was followed by a slower decline that almost reached the baseline at 2.5 min (*n* = 6 cells, 1 patient) (Figures 3(c) and 3(d)).

### 3.3. Secretory Cells in Culture

A total of 160 mature 21 day cultures of secretory cells, to study secretion and mucin expression, were obtained from 98 patients. Cells grown in flasks and 4-well chambers formed a confluent monolayer within 7-8 days after being seeded and progressively differentiated, forming a secretory monolayer between days 14 and 21 (Figure 1(c)). Microscopic observation during the period of cell growth, showed cell adherence to the flask and also to the coverslip coated with collagen used in the 4-well chamber. During the third week of culture, cells cover 50–70% of the growing plate surface. 

Spontaneously, the secretory cells expelled material in a spherical shape which displayed rapid emergence (2–20 s) from the cell surface (see arrow in Figure 1(c)). In these cultures we also observed ciliary cells predominantly in the first two weeks of culture.

To demonstrate the presence of intracellular mucin in cells, with secretory activity (Figure 1(c)), primary cultures were fixed when an active secretion was observed and processed for immunofluorescence using MUC5AC and MUC5B antibody (Figure 1(d)). Both mucins were subcellulary distributed in granules (arrow in Figure 1(d)). These observations indicated that cell culture conditions allowed for the development of cultures enriched in secretory cells from the respiratory epithelium. 

**Figure 4:**
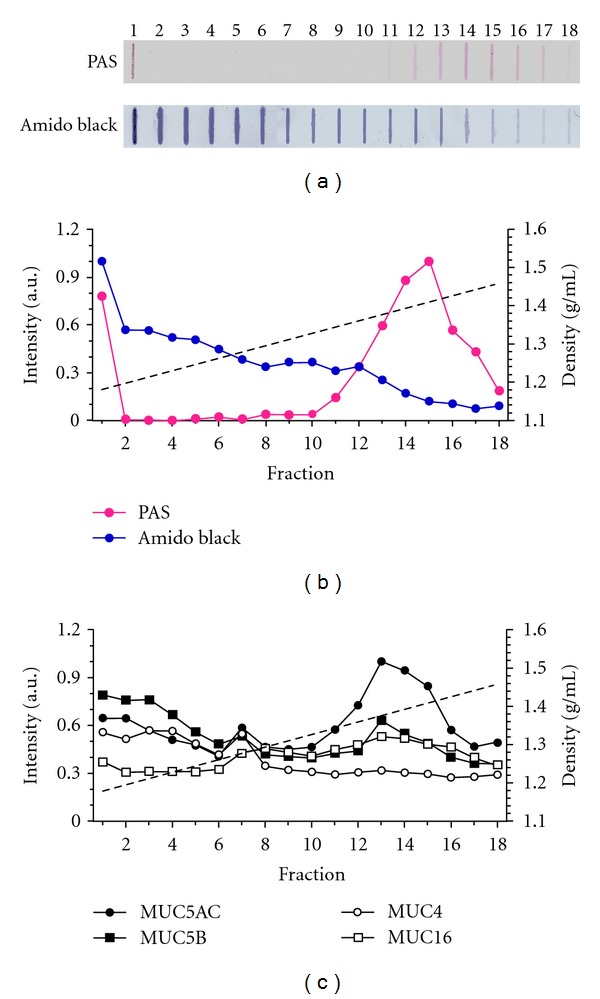
Distribution of mucins in isopycnic gradient. Identification of mucins from conditioned medium produced after 21-day primary cultures of adenoid epithelium, and obtained after centrifugation in an isopycnic gradient. (a) Slot blot of the glycosylated material made dye and proteins visible with PAS and was evidenced by Amido black, for the 18 fractions of the gradient. Low density fractions correspond to higher content of proteins while high density fractions to glycoproteins. (b) Analysis of the distribution of glycosylated material (red circles) and proteins (blue circles) from (a). The straight line corresponds to the lineal regression (*r* = 0.99) of the density of the fractions (g/mL) (b) and (c). (c) Mono and polyclonal antibodies were used to evidence the presence of MUC5B (square fill), MUC4 (circles empty), MUC16 (square empty) and MUC5AC (circles fill). The normalized pixels, in all cases, correspond to the intensity of pixels under the curve, horizontally quantified, divided by the average value of intensity for each condition.

*Mucins Secretion in Culture. *The analysis of the fractions obtained from CsCl/GdmCl gradient, showed a distribution of proteins along the gradient, where the most intense reactions were observed in the first 13 fractions for amido black (Figure 4(a)). PAS positive material was observed between fractions 11 and 18, with a maximum colorimetric intensity between fractions 14 and 15 for PAS reaction (Figure 4(b)). The pattern of secreted PAS positive material corresponds to glycosylated molecules which are found in a range of density between 1.331 and 1.475 g/mL (Figures 4(a) and 4(b)), as previously described for mature mucins in other respiratory culture systems [37].

Slot blot using specific antibodies for mucins forming gel and bound to membrane, showed the presence of different mucins: MUC5AC, MUC5B, MUC4, and MUC16 in the conditioned medium (Figure 4(c)). The highest intensity label for the antibody and PAS reaction corresponds to MUC5AC, mucin known to form polymer gel structure in the airways [32]. In addition, in the immunodetection of the protein core of mucins (apomucin) forming gel as MUC5AC and MUC5B, only MUC5AC was present, mainly in the carbohydrate enriched fractions (Figure 4(b)). The membrane mucins MUC4 and MUC16 were present in the cultures, but not glycosylated and in smaller amount (Figure 4(c)). 

**Figure 5:**
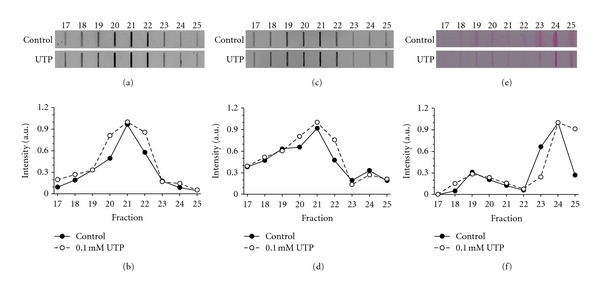
Characterization of MUC5AC from conditioned media in the presence or absence of UTP. Mucins present in the 21-day conditioned culture mediums were concentrated via centrifugation in CsCl 1.5 g/mL for 16 h and 20 min. Fractions 17–25 are shown from the conditioned medium under conditions control (*n* = 6 conditioned medium) (circle fill), and UTP (*n* = 5, conditioned medium in the presence of 100 *μ*M UTP for 22 h) (circle empty). (a) Immune slot blot for MUC5AC. (c) Immune slot blot for sialic acid using a biotinylated lectin. (e) Slot blot for PAS reaction. (b), (c) and (d) The normalized pixels, in all cases, correspond to the intensity of pixels under the curve, horizontally quantified, divided by the average value of intensity for each condition.

*Mucin Detected in Conditioned Media of Cultures in the Presence or Absence of UTP. *Previous studies have shown that the addition of UTP to secreting cells in culture stimulates mucin secretion. The profile distribution of MUC5AC, the most abundant mucin obtained from the cultures, in the presence or absence of 0.1 mM UTP for 22 h (Figure 5) was analyzed. Signal intensity showed a Gaussian distribution for MUC5AC and did not show any significant differences between the control and UTP treated cultures (Figures 5(a) and 5(b)). To verify the presence of sialic acid that faces the end of *O*-linked carbohydrate of mucins chains, in different fractions from the CsCl gradient, lectin that recognize sialic acid residues in glycosylated ramifications was used and the sialic acid distribution and intensity in the same fractions (17–25) previously analyzed for MUC5AC was investigated. Sialic acid distribution and intensity patterns (Figures 5(c) and 5(d)), were found to be very similar in the presence or absence of UTP 0.1 mM. In both cases, the sialic acid peak intensity is located in fraction number 21. This peak coincides with the peak of MUC5AC in Figure 5(b), and indicates that UTP did not affect the sialic acid distribution pattern for MUC5AC secreted in cultures. The analysis of glycosylated material identified on slot blot using PAS reaction (Figures 5(e) and 5(f)), in the same experimental conditions, showed a PAS positive material toward the end portions of the fractions, corresponding to fractions 18–25. The peak of the PAS intensity reaction for the control group was observed within fractions 23-24; for the UTP treated group mainly is restricted to fraction 24. However, in both cases the increase in the carbohydrate content corresponds to the higher density fractions expected for mucins. 

**Figure 6:**
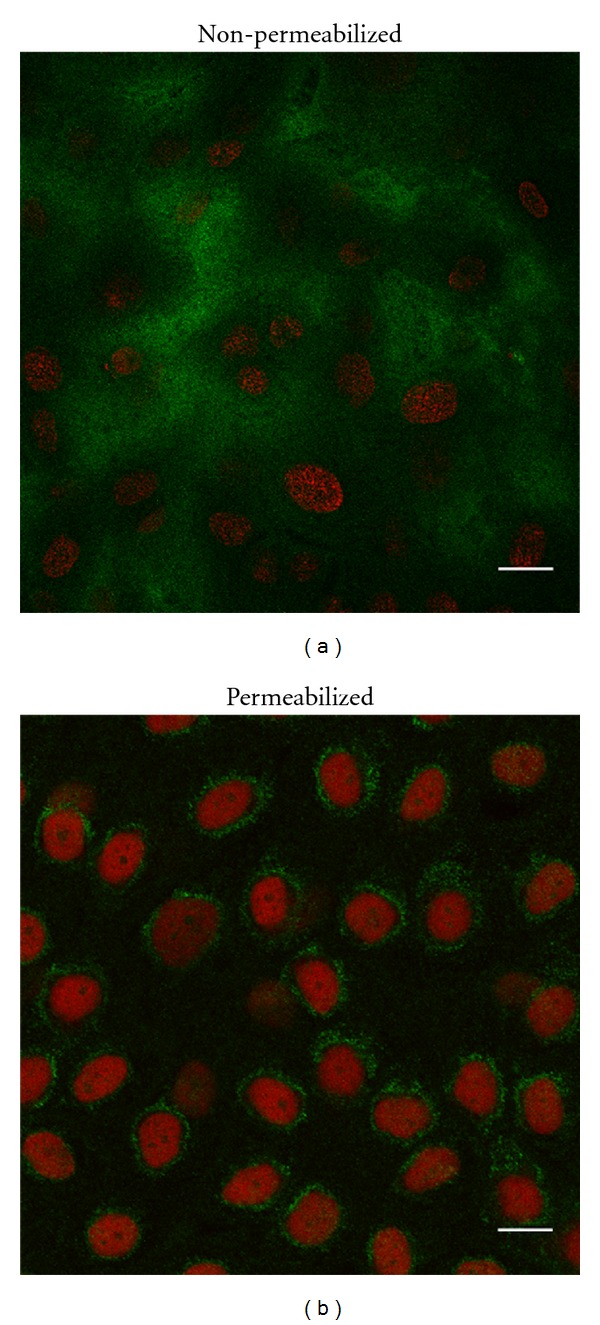
Microphotograph of a confocal microscopy image of MUC5AC in cultures of secretory cells from adenoid epithelium. Cultures of secretory cells were fixed by means of 2% PFA and marked with two different antibodies against MUC5AC (green). (a) For the nonpermeabilized cells, antibody MAN5AC was used. (b) For the permeabilized cells, the polyclonal antibody MUC5AC (from Dr. Ho) was used. A diffuse label covering the cells surface was observed for the nonpermeabilized cells, as well as a discrete granular distribution for the permeabilized cells. Nuclei were stained with propidium iodide. Bar = 20 **μ**m.

*Detection of Mucin MUC5AC in Permeabilized and Nonpermeabilized Cells. *To establish whether the secreted material observed in phase contrast microscopy (Figure 1(d)) contained mucins, permeabilized and nonpermeabilized cells were used for immunofluorescence against MUC5AC. It was established that in nonpermeabilized cells, the immunoreactive label against MUC5AC is diffuse, and covers most of the surface of the cell culture (Figure 6(a)). In contrast, the label for the permeabilized cells was discrete, corresponding to a granular distribution surrounding the nucleus (Figure 6(b)). 

## 4. Discussion

 In the present study, it has been found that cultures of ciliated and secretory cells from the upper human airway, derived from adenoid tissue explants, constitute a reproducible and reliable experimental model to study the control mechanisms of ciliary activity and mucin secretion. The culture protocol of this experiment allowed ciliated cells to be maintained with spontaneous ciliary activity, and a basal average CBF of 10.7 Hz, similar to previous reports from upper airway cultures [2]. In addition, from the same tissue, it was possible to establish a cell culture protocol to obtain secretory cells with spontaneous secretory activity. The immuno slot blot analysis demonstrated the presence of different glycoproteins, including the most important mucins MUC5AC and MUC5B which contribute to the structure of the airway mucus (Figure 4(b)) [38]. Biochemical characterization of MUC5AC, the most prominent mucin collected from conditioned medium, showed fractions of higher density glycoproteins with elevated levels of sialic acid (Figures 5(a)–5(d)) and other sugars detected with PAS reaction (Figures 5(e)–5(f)). These findings provide evidence that adenoid epithelium in culture have morphological characteristics similar to the airways epithelium *in vivo.* In these cultures, ciliated cells showed coordinated activity and secretory cells released mucins with an heterogeneous distribution of sugar and sialic contents, representative of the biochemical profile mucins from the airways.

Adenoid tissue explants have been used as a common method to study ciliary activity *in vitro* [22, 39–41]. Basically, explants are kept in saline solution or buffer for CBF measurements shortly after surgery. With this method CBF and ciliary signal, are exposed to mechanical trauma and inflammatory mediators released during removal of the tissue sample. Furthermore, the simultaneous presence of ciliated cells and secreting cells in the tissue can make it difficult to determine if the specific effect of an experimental treatment is related to either one of the two cell types present in the explants. Our technique allowed for procurement of enriched monolayers ciliated cells after 9 days in culture, and mucin-secreting cells after 21 days in culture. The culture technique used in this study required tissue from a single patient and allowed for the growth of each cell type separately and for the study of the control mechanisms of each cell function independently. The large size of the adenoid tissue is a comparative advantage relative to samples from nasal mucosa (turbinates, polyps), since it increases the number of cultures to be obtained from one patient [42]. Another advantage is that adenoids usually come from healthy patients, whose epithelium has not been under chronic stimulation of inflammatory cytokines or chronic infections. Also, since adenoids are usually discarded, obtaining this sample does not add any extra morbidity to patients, while taken a biopsy from turbinates or septal mucosa in patients without a medical indication can cause epistaxis. 

In ciliated cell cultures, a cell monolayer is formed around the epithelial explant, which were the cells used in this study, and not the cells present in the explant. The exact origin of these cells cannot be established, however. One possibility is that they migrate from the explant and adhere to the collagen matrix, constituting a cell monolayer away from the original explant, or undifferentiated cells from the explant migrate to form the monolayer, and after that differentiate into ciliated or secretory cells. The former hypothesis seems more likely possible, considering the brief time needed to obtain active and mature cells, compared to the ALI culture system, where mature cells are obtained after 30 days [17–19]. In order to understand this finding, future studies will be required.

As the cells of our cultures grow under a liquid phase, they are flat, so this model probably does not allow for studies in which cell polarization is important. In these cases, ALI cultures are more suitable. 

Within cultures, basal CBF followed a normal distribution in the range between 5.9 Hz and 17.5 Hz as previously described for other ciliated epithelium [2]. It can be established that CBF do not vary within cultures of different days of growth, providing conditions to perform equivalent experiments in cells grown after different days and increasing the yield of cultures obtained out of a single biopsy.

Cultures of ciliated cells have been used to characterize the response of CBF to different chemical signals and the respective transduction pathways involved in ciliated cell activation. The response obtained to different ATP concentrations in the model presented here is comparable to the response described by other authors [12, 38, 43, 44]. A 27.2 ± 2.7% increased in CBF over the baseline in response to 10 **μ**M ATP was observed. In human trachea, cultures with a same final ATP concentration produced a 37 ± 4% increase in CBF [12]. ATP produces a rapid increase in CBF, associated with an increase of intracellular calcium levels [44]. In the cells used in this study, intracellular calcium levels rapidly increase followed by a slow decay to a baseline after 2.5 min of ATP stimulation. This response is any different from the previous changes in intracellular calcium after ATP stimulation in other ciliated epithelium [12]. The functional response of ciliated cells to ATP, UTP, and adenosine observed in the present study validates the importance of these nucleotides in the control mechanism of the ciliary activity, independently of the region of the airway epithelium.

Secretory cells cultures were recognized by their spontaneous secretory activity and the secreted mucins were MUC5AC and MUC5B (Figure 4(c)) [14, 45]. The histological characterization of the epithelial of the adenoid tissue indicated the presence of cells containing inside vesicles PAS-positive. Also, it was possible to observe that MUC5AC preferentially disposed, but not exclusively, on the apical epithelial border. These results suggest that the epithelial cells used to develop cultures were secretory cells, with mucin secretion. The difference between PAS stain and MUC5AC signal could be explained because the MUC5AC antibody recognizes the apomucin, epitope which may not be exposed in mucins contained within the secretory vesicles. 

Secretory cells grown in culture with the protocol described in this study, identified cells with spontaneous degranulation, where the material secreted was observed emerging from the cell surface, in fact, rapidly expanded (5 to 20 s), forming spherical structures (arrows in Figure 1(c)) which reached diameter similar to those observed previously in other biological models [46, 47]. The content of the secretory vesicles—water, ions, and other molecules present in the luminal surface of the epithelium—will form the mucus layer, characteristic of the airways surface [13]. When the secretory cells were fixed and processed by double immunofluorescence, it was also observed that MUC5AC and MUC5B organized in vesicles inside the cells (arrow in Figure 1(d)). It was not determined which of the two mucins was more abundant within the vesicles of the cell culture. However, preliminary data using colocalization distribution of both mucins indicates that the density might be diverse within secretory vesicles. 

The biochemical characterization of the collected conditioned medium from cell culture showed a distribution of proteins and glycosylated material which corresponds to a typical mucin profile [37]. This reaction between the dye and the proteins is greater (because of its low density) in the absence of glycosylated material (high density) (Figure 4(a)). The immuno slot blot analysis demonstrated that the presence of different mucins including the most important ones, MUC5AC and MUC5B, contribute to the structure of the airway mucus (Figure 4(b)) [38]. The use of mono- and polyclonal antibodies against MUC5B shows a weak signal compared to MUC5AC (Figure 4(c)), similar to the observations previously described in polarized primary cultures of human tracheal epithelium [37]. Probably, the extraction technique of adenoid epithelium favored the growth of mucosal cells from the epithelial surface, which produced abundant MUC5AC mucin, unlike MUC5B, the main product of the cells in submucosal glands [14]. 

MUC5AC was synthesized and secreted by the epithelial cells in mature primary cultures of adenoid tissue in two different states, a mature glycosylated MUC5AC, fractions 12−16 (Figure 4(c)), and a state of unglycosylated MUC5AC, fraction 1–3 (Figure 4(c)). The isopycnic density gradient of CsCl/GdmCl serves the separation of mucins according to their flotation density. The difference between flotation densities for each population of MUC5AC is due to both a decrease in the sugar content of MUC5AC and probably also to differences in the polymeric state [48]. These biochemical characteristics explain the broad pattern of distribution present in the slot blot analysis of the conditioned medium. 

To further characterize the presence of other mucins in the culture, MUC4 and MUC16 membrane mucins were tested, which are also present in these cultures; however, they are not glycosylated and were observed in a less significant quantity.

Conditioned culture medium, which contained mucins after 21 days in culture, were concentrated by centrifugation in CsCl 1.5 g/mL and analyzed by slot blot. The pattern of distribution of MUC5AC observed in Figure 4, did not change. This result indicated that both instances of centrifugation (14 h at 202.048 ×g versus 72 h at 171.500 ×g) separated MUC5AC in similar patterns, obtaining a population shifted toward the end portions of the fractions of the tube (fractions 20–22 in Figures 5(b) and 5(d)). This result indicated that a less timed and higher speeded centrifugation at could be more cost/effective for mucins separation.

The purinergic agonists have been reported to cause degranulation of secretory cells and induce the release of glycoproteins [33], so whether a 22 h treatment with 0.1 mM UTP favored mucin secretion or not was studied. The analysis of culture medium concentrated by centrifugation in CsCl 1.5 g/mL, in the presence or absence of UTP, indicated that fractions enriched in apomucin 5AC contained carbohydrates like sialic acid (Figures 5(b) and 5(d)), with a similar pattern distribution and immunoreactive intensity (Figures 5(a)–5(d)). A similar result has been observed in bronchial tissue sections for MUC5AC, incubated with UTP for short periods of time [49].

It was not observed, however, any effect of UTP on the secretion increase of MUC5AC, as has been previously described in primary cultures from human tracheobronchial epithelial cells, where the detection was done 1 h after UTP treatment [50]. Possible explanations for this disagreement on the effect of UTP might be related to the region of the respiratory tract from where the cells are obtained. Furthermore, the time of exposure of cells to UTP and the type of epithelial cell culture seem to determine the response, since UTP increased mucin secretion in polarized cells in culture [33, 50]; this was not observed in tissue sections [49].

Although there was no biochemical difference between the control and UTP treated cultures, differences in the peak intensity between the slot blot analysis of MUC5AC, sialic acid and glycosylated material were evidenced (Figures 5(b), 5(d) and 5(f)). These findings could be explained by the low sensibility of PAS reaction, when detecting the presence of sialic acid in fraction 21, where the mucin MUC5AC is present. It is also possible that other monosaccharides, associated to other molecules are detected by a PAS reaction in higher fractions of the slot blot (Figure 5).

The immunological characterization of the secretory cells culture provided evidence of mucin MUC5AC, glycoprotein located both on the cell surface for nonpermeabilized cells (Figure 6(a)) and within the secretory vesicles of the majority of the permeabilized cells present in the culture (Figure 6(b)). These observations support the feasibility of biochemical studies using this secretory cell system.

Biochemical analysis of secretory material collected from the adenoid human cultures provides evidence of the complex regulation of glycoprotein synthesis, including sialic acid and sugar content, and the potential use of this culture technique to characterize the molecular structure of mucins in the airways epithelium. 

## 5. Conclusion

 Culture of ciliated and secretory cells from adenoid explants is a reproducible experimental model, in which it is possible to observe cells with functional characteristics similar to those of the epithelium *in vivo*. The functional response of ciliated cells to nucleotides such as ATP or UTP, coupled to intracellular calcium changes, provides a potentially powerful model to study the signal transduction mechanisms involved in the control of ciliary activity. The collection of mucins from cultures suggests a model for further biochemical characterization of molecular structure of glycoprotein in different experimental conditions. This work describes two culture techniques and constitutes an excellent tool to develop research protocols on human secretory and ciliated cells studies in pathological and physiological conditions.
